# Survival Analysis of Demographic Factors Associated With 5+ Year Survival of Pancreatic Carcinoma

**DOI:** 10.7759/cureus.13032

**Published:** 2021-01-31

**Authors:** Chris Anama-Green, Megan Quinn

**Affiliations:** 1 Health Science and Education, University of the People, Pasadena, USA; 2 Education, University of the Cumberlands, Williamsburg, USA; 3 Epidemiology and Public Health, East Tennessee State University, Johnson City, USA

**Keywords:** cancer, pancreatic cancer, carcinoma, survival, seer, national cancer database and seer analyses

## Abstract

Background

Although pancreatic cancer incidence is low at 13.1 per 100,000 people, this cancer is difficult to treat and carries a poor 5-year survival rate. Additionally, pancreatic cancer survival rates vary disproportionately based on age and race. The objective of this study was to evaluate the association between 5-year survival of pancreatic cancer and the basic demographic factors age, race, and sex.

Methods

Data were retrieved from the National Cancer Institute's Surveillance, Epidemiology, and End Results (SEER) 18 database, spanning from 2000-2017, using SEER*Stat. SPSS was used to calculate descriptive statistics for vital status, age, race, and sex. Odds ratios with confidence intervals were calculated using Epi Info. Case data were used to conduct survival analysis by age, race, and sex using OriginPro.

Results

Out of a total of 118,581 cases, 79.3% were White (n = 106,887), 12.5% were Black (n = 16,866), 7.4% were Asian or Pacific Islander (n = 9,960), 0.6% were American Indian/Alaskan Native (n = 792), and 0.2% were unknown race (n = 321). The odds ratio (OR) of dying before reaching 5+ survival was lowest for the Asian or Pacific Islander group (OR = 0.70, 95% CI: 0.66 - 0.74), followed by the group of Black patients (OR = 1.07, 95% CI: 1.02 - 1.13), the White patients group (OR = 1.12, 95% CI: 1.08 - 1.17), and the American Indian/Alaskan Native group (OR = 1.12, 95% CI: 0.89 - 1.40). The largest age group was 65-69 years old, comprising 14.7% (n = 19,866) of the dataset. Probability of 5+ year survival for pancreatic cancer patients was highest for the age group 15-19 years (n = 74). In general, 5+ year survival probability declined with age. Risk of death before reaching 5+ year pancreatic cancer survival was slightly higher in men (OR = 1.03, 95% CI: 1.00 - 1.07), who comprised 50.9% (n = 68,628) of the dataset.

Discussion

Findings from this study corroborate differences by age, race, and sex discussed in the literature. Differences in survival rates by race depart from some findings in literature documenting no significant differences in treatment outcome by race. Controlling for age in a future study in both race and sex survival probability analyses may be helpful. Further, stratifying by sex in survival probability analysis by race would be illuminating. In addition to survival analysis, regression modeling would be a useful next step.

## Introduction

As of 2020, pancreatic cancer comprised 3.2% of all new cancer cases in the United States. However, it was associated with 7.8% of all cancer deaths in the United States. The age-adjusted incidence rate of new pancreatic cancer cases in the United States is 13.1 per 100,000. Lifetime risk of being diagnosed with pancreatic cancer in the United States is 1.6%. Although the incidence of pancreatic cancer is relatively low in the United States as compared with other cancers, its 5-year survival rate from 2010-2016 was only 10% [1].

Despite significant advances in cancer treatment in the past 30 years, pancreatic cancer remains challenging to treat. Early diagnosis is associated with comparably more successful treatment [2]. Unfortunately, pancreatic cancer is not often diagnosed until later stages, which can complicate successful treatment and compromise a patient's likelihood of 5+ year survival. Patients with pancreatic cancer often receive this diagnosis later in the disease progression because early-stage pancreatic cancer often begins without noticeable symptoms. As symptoms of pancreatic cancer emerge, they are generally similar to those of other non-cancerous health problems including pancreatitis [3]. When finally diagnosed, what began as a localized pancreatic tumor may have already metastasized [2,3].

Due to the challenges associated with diagnosing and treating pancreatic cancer, pancreatic cancer carries a low 5+ year survival rate in the United States [1,4]. As such, literature reports that this cancer's average 5+ year survival rate has not improved at a rate comparable with cancers of other sites [2]. Adding further complication, previous studies suggest that significant differences in survival rates are present among pancreatic cancer patients based on patient demographics [5]. Pancreatic cancer survival rate disparities in the United States cannot be fully explained based on biological factors or other non-demographic factors [4,5].

The United States cancer data is available from the National Cancer Institute's Surveillance, Epidemiology, and End Results (SEER) Program. Depending on the databases selected for analysis, SEER data includes up to 11,135,914 total malignant and in situ cases [1]. This data has been used to study cancer trends and survival in numerous published studies [1,3,6]. SEER database can be used to analyze pancreatic cancer cases based on demographic data or to export relevant variables for analysis with an external tool. Using SEER data [1], this study evaluated the association between 5+ year survival of pancreatic cancer and the demographic factors of age, race, and sex.

## Materials and methods

SEER*Stat [1] was used to retrieve cancer incidence data (SEER 18: 2000-2017) from the National Cancer Institute. Only cases with pancreatic carcinoma [7] were included with the relevant demographic variables age, race, and sex. SEER grouped patient ages into fourteen brackets of five years each for ages 15 years through 84 years and one category for 85+ years. Races assigned to patients in the SEER dataset included American Indian / Alaskan Native, Asian / Pacific Islander, Black, White, and "unknown" [1].

IBM SPSS [8] was used to generate descriptive statistics for age, race, and sex from case data. SPSS [8] crosstabs were also used to generate 2x2 table data for race and sex. This information was used to conduct odds ratio analysis using Epi Info [9]. Odds ratios (OR) were calculated with confidence intervals, to evaluate odds of death before reaching 5+ year survival of pancreatic carcinoma. Cases with "unknown" race (n = 321) were excluded for analyses by race. OR calculations were completed for each individual race as compared with "all" other races in the dataset. Additional analyses were completed to compare the Asian and Pacific Islander group, which was the group with the highest odds of reaching 5+ year survival, and other race groups. OR values were calculated to compare survival odds for this group with American Indian / Alaskan Native, White, and Black groups.

Survival analysis was conducted using OriginPro [10]. Survival distributions based on age, race, and sex were calculated using the Kaplan-Meier (product-limit) survival analysis method with months of survival as the time variable, vital status as the censor variable, and a demographic factor (i.e., age, race, or sex) as the grouping variable.

## Results

Descriptives

The dataset contained 49.1% (n = 66,198) female cases and 50.9% (n = 68,628) male cases for a total of 134,826 pancreatic cancer cases. Vital status was "alive" for 12.1% (n = 16,265) of the recorded cases and "deceased" for the remaining 87.9% (n = 118,561) of cases.

The lowest number of cases was present in the 15-19 years age group, which represented 0.1% (n = 74) of the dataset. The age group with the highest number of cases was 65-69 years, representing 14.7% (n = 19,866) of the dataset. Frequency increased for each age group beginning with the 15-19 years age group and ending with the 65-69 years age group. Beginning with the 20-24 years age group the relative frequency of pancreatic cancer doubles with each successive age group, ending after the 40-44 years group. From the 50-59 age group through the 65-69 age group relative frequency continues to increase. At ages 70-74 the number of pancreatic cancer cases included in the dataset begins to decline with each successive age group (Table 1).

**Table 1:** Descriptive statistics for age.

Age group	Frequency	Percent
15-19 years	74	.1
20-24 years	114	.1
25-29 years	227	.2
30-34 years	518	.4
35-39 years	1146	.8
40-44 years	2509	1.9
45-49 years	5390	4.0
50-54 years	9745	7.2
55-59 years	14473	10.7
60-64 years	18174	13.5
65-69 years	19866	14.7
70-74 years	19340	14.3
75-79 years	17696	13.1
80-84 years	14037	10.4
85+ years	11517	8.5
Total	134826	100.0

The majority of cases included in the dataset were White, comprising 79.3% (n = 106,887) of the dataset. This was followed by Black cases, representing 12.5% (n = 16,866) of all cases. Asian or Pacific Islander accounted for 7.4% (n = 9,960) of the dataset. Finally, American Indian/Alaskan Native comprised 0.6% (n = 792) of the dataset. A total of 321 cases had an unknown race (Table 2). These unknown cases were excluded in the survival analysis based on race.

**Table 2:** Descriptive statistics for race.

Race	Frequency	Percent
American Indian/Alaska Native	792	.6
Asian or Pacific Islander	9960	7.4
Black	16866	12.5
Unknown	321	.2
White	106887	79.3
Total	134826	100.0

Survival analysis

**Figure 1:**
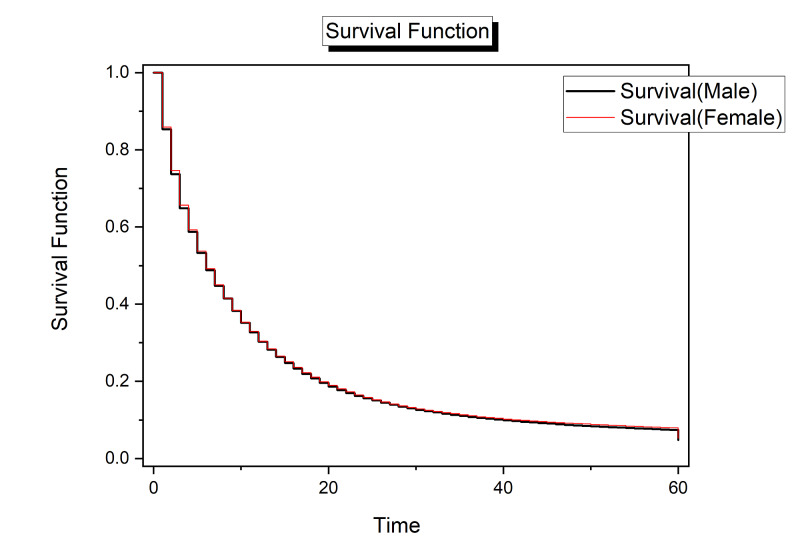
Survival analysis for pancreatic cancer by sex. Time (x-axis) is given in months.

Survival analysis based on sex suggested that females had measurably increased odds of survival earlier in disease progression. That is, overall females were more likely to survive in the initial months following diagnosis during at least the first 10 months. Beyond the first 10-month survival rates were nearly indistinguishable between men and women (Figure 1).

**Figure 2:**
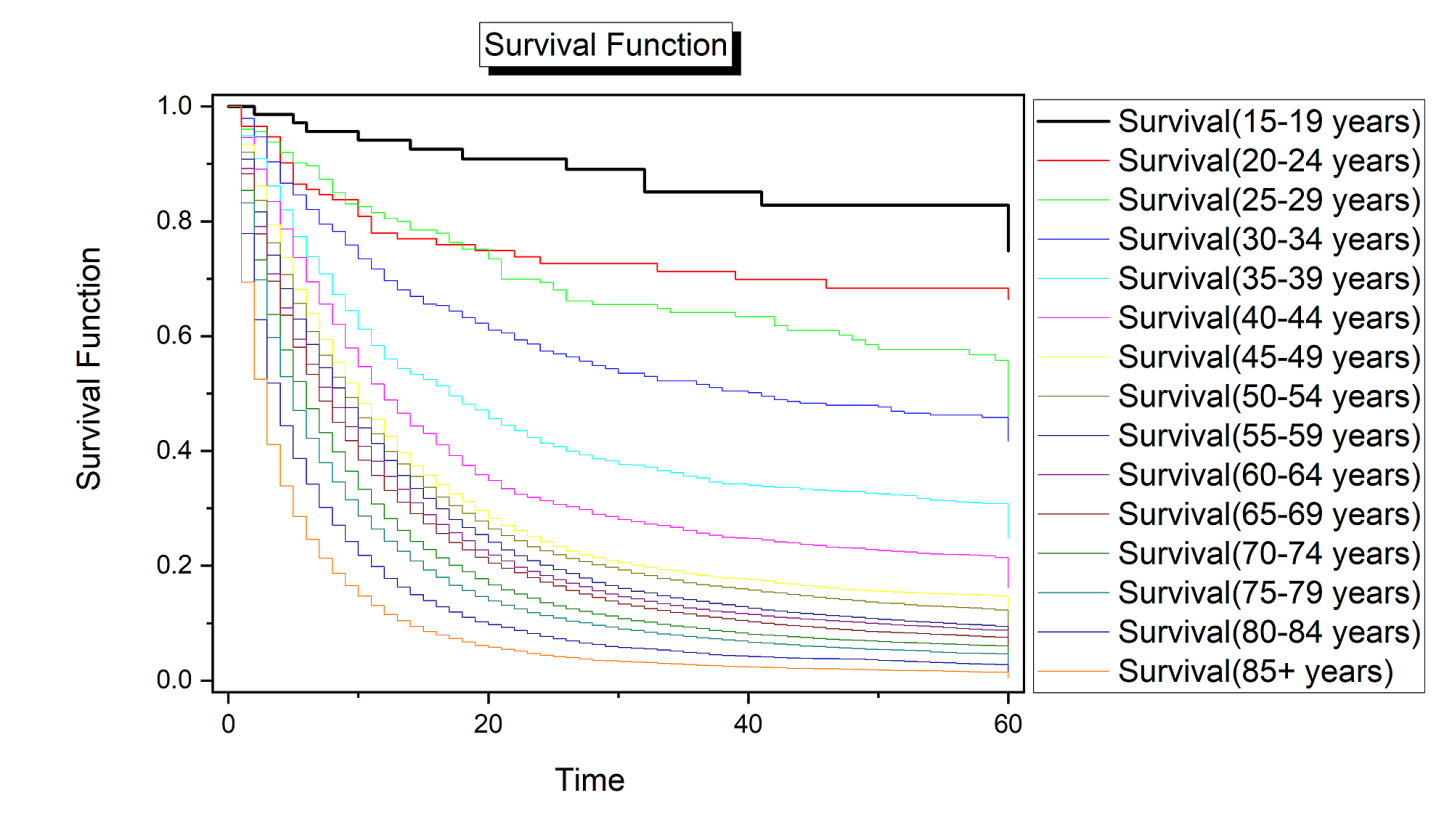
Survival analysis for pancreatic cancer by age. Time (x-axis) is given in months.

With few exceptions, survival odds were higher for younger age groups across the timespan of disease progression. The age group 25-29 years had higher survival odds earlier in the survival function than the 20-24 years age group. This trend reversed at approximately 20 months. From that point to the end of the timespan of the survival function, age category was a strong predictor of survival with younger age groups having significantly higher odds of survival than older age groups (Figure 2).

**Figure 3:**
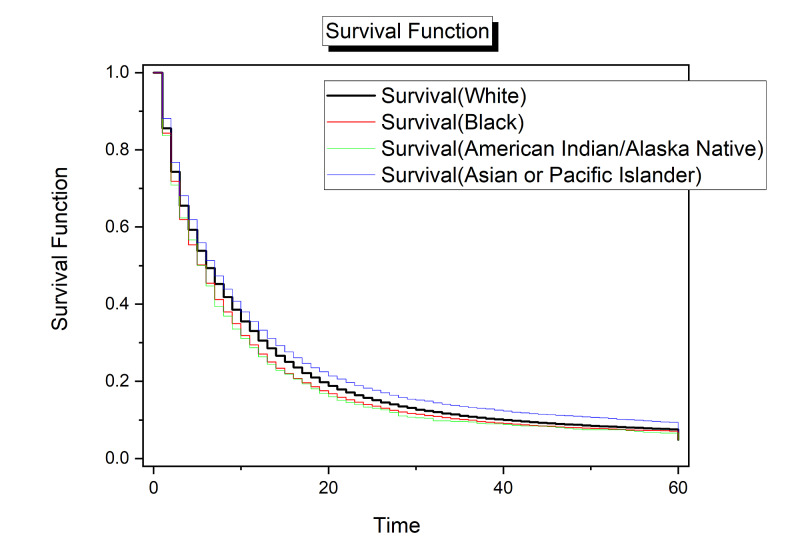
Survival analysis for race. Time (x-axis) is given months.

Survival rates were similar for all cases at the beginning of the survival function, but trended apart as time increased. The highest survival rates across the function were Asian or Pacific Islander cases. Black cases had lower survival rates than White cases at every step of the survival function. Survival rates of American Indian/Alaskan Native cases trended below all other races as time increased (Figure 3).

Odds-based analysis

Analysis based on sex suggested that the risk of death before reaching 5+ year survival of pancreatic cancer was slightly higher for men (OR = 1.03, 95% CI: 1.00 - 1.07). While this small difference was significant, the risk difference is not large enough to suggest that major differences in risk are present based on sex. 

The Asian/Pacific Islander group had the lowest odds of dying from pancreatic cancer before reaching 5+ year survival as compared with all other races. The group with Black patients followed. White and American Indian/Alaskan Native had the highest odds ratios. In comparison with the Asian/Pacific Islander group, the Black group (OR = 1.48, 95% CI: 1.38 - 1.59) and the White group (OR = 1.43, 95% CI: 1.25 - 1.51) were each 5% more likely to die before reaching 5+ year pancreatic cancer survival. The American Indian / Alaskan Native group (OR = 1.56, 95% CI: 1.24 - 1.96) was 6% more likely to die before reaching 5+ year survival as compared with the Asian/Pacific Islander group (Table 3).

**Table 3:** Odds of dying before reaching 5+ year pancreatic cancer survival (by race).

Race	OR
American Indian / Alaskan Native	1.12 (95% CI: 0.89 – 1.40)
Asian / Pacific Islander	0.70 (95% CI: 0.66 – 0.74)
Black	1.07 (95% CI: 1.02 – 1.13)
White	1.12 (95% CI: 1.08 – 1.17)

## Discussion

Results of five-year age-standardized survival by race are consistent with reported incidence rates [1] but contrast with some findings in the literature. A 2013 study found no significant difference in survival rates by race in patients in the study population [11]. However, this study may not be comparable with the broader experience of pancreatic cancer patients in the United States because it was conducted with a population traced in the US Department of Defense facilities [11]. Findings from other studies highlight differences in treatment outcomes based on race for pancreatic cancer cases and for patients with cancer in other sites [2,4-6].

A key limitation of the selected dataset is the coding of Hispanic and Latino patients. The dataset in the current study does not denote Hispanic and Latino ethnicity. The lack of this distinction could influence the results, as Hispanic and Latino patients are included in the study but are only recorded in the current dataset by their self-reported race. This also precludes analysis of Hispanic and Latino survival rates in comparison with other race categories. Inclusion of Hispanic and Latino ethnicity classification would lend higher resolution to analyses by race in future studies.

The SEER databases do not cover the entire United States population. As such, SEER studies sometimes make the assumption that the dataset is representative of the nation's cancer outcomes [12]. However, with coverage of 35% of the U.S. population [1], it is important to interpret the data from this and other SEER studies in context [1,12].

Differences in survival probabilities by age in this study met the expected trend of declining probability of survival as age increases. In the study dataset, this trend emerged beginning in the age group 25-29 and strengthened as age increased. More research is necessary to better understand the role of age in pancreatic cancer survival beyond 5 years. Relating age with biological factors and behavioral risk factors could improve our ability to predict pancreatic cancer risk and likelihood of 5+ year survival for those diagnosed with pancreatic cancer.

This study addressed age, race, and sex separately in survival probability analysis. Controlling for age in a future study in both race and sex survival probability analyses may be helpful. Further, stratifying by sex in survival probability analysis by race would be illuminating. In addition to survival analysis, regression modeling would be a useful next step. Continued research on the role of lifestyle factors, genetics, and other non-demographic factors remains crucial as we investigate risk factors associated with pancreatic cancer and improve our methods to promote earlier diagnosis.

## Conclusions

Significant differences in 5+ year survival rates of pancreatic cancer were observed based on race, age, and sex. Asian and Pacific Islander patients had the highest survival probability followed by White patients, Black patients, and American Indian/Alaskan Native patients. Significant differences were present by age in terms of the frequency of pancreatic cancer diagnosis and the frequency of 5+ year survival. In general, the likelihood of 5+ year survival decreased as age increased. With respect to sex, men had significantly higher odds of dying before reaching the 5-year survival mark, as compared with women. However, this difference was slight and did not suggest drastic differences in risk of dying before reaching 5+ year survival based on sex. More research is needed to improve our ability to achieve earlier diagnosis of pancreatic cancer and to improve treatment outcomes.
